# Combination effect of meropenem and antifungals against Escherichia coli-Candida albicans dual-species biofilms in vitro and in vivo using a silkworm model

**DOI:** 10.1099/jmm.0.002061

**Published:** 2025-09-09

**Authors:** Sanae Kurakado, Kakeru Yasuda, Yasuhiko Matsumoto, Takashi Sugita

**Affiliations:** 1 Department of Microbiology, Meiji Pharmaceutical University, Tokyo, Japan

**Keywords:** biofilm, *Candida albicans*, combination drug effect, *Escherichia coli*, silkworm model, tolerance

## Abstract

**Introduction.** Biofilms are a primary form of device-associated infections and typically exhibit high tolerance to antimicrobial agents. In biofilms formed by multiple microbial species, microorganisms may show even greater tolerance, complicating treatment. There is evidence that meropenem (MEPM) tolerance in *Escherichia coli* is increased in dual-species biofilms with *Candida albicans*, and effective treatments have not been established.

**Hypothesis/Gap Statement.** If the presence of viable *C. albicans* increases the MEPM tolerance of *E. coli* in mature biofilms, then the killing of *C. albicans* will attenuate the MEPM tolerance of *E. coli*.

**Aim.** We evaluated the effectiveness of various antifungal combination treatments against dual-species biofilms of *E. coli* and *C. albicans in vitro* and *in vivo*.

**Methodology.** The reduction in the number of viable cells in dual-species mature biofilms formed by *E. coli* and *C. albicans* was evaluated after treatment with a combination of antifungal drugs (fluconazole, amphotericin B and micafungin) and MEPM. In addition, the *in vivo* effects of combination therapy were assessed using a silkworm biofilm infection model.

**Results.** The combination of amphotericin B and MEPM reduced the viable cell counts of both *E. coli* and *C. albicans* within dual-species biofilms. In contrast, the combination of fluconazole and MEPM did not reduce the viable cell count of either species, whereas the combination of micafungin and MEPM reduced *C. albicans* only. The reduction in viable *C. albicans* counts by micafungin was less than that by amphotericin B, suggesting that micafungin did not affect the tolerance of *E. coli*. The combination of amphotericin B and MEPM also reduced the viable cell counts of both *E. coli* and *C. albicans* in the *in vivo* model.

**Conclusion.** These findings suggest that the combination of amphotericin B and antibacterial agents is a potential treatment option to reduce the *C. albicans*-induced bacterial tolerance for catheter-related infections involving *C. albicans* co-infection.

## Introduction

Catheter-related bloodstream infections (CRBSIs) can lead to severe complications in major organs, including bacterial endocarditis, pulmonary abscesses and infectious embolism, often accompanied by sepsis or septic shock [1]. It has been reported that 21% of bloodstream infections (BSIs) in intensive care units are catheter-related [2], with the presence of a medical device such as catheters being a risk factor for BSI [3]. The crude excess mortality rate for CRBSIs is estimated to be 23.6% [4], and the mortality rate of BSI rises sharply if appropriate antimicrobial therapy is delayed.

Medical devices, particularly catheters, serve as substrates for biofilm formation. Biofilms are composed of aggregated micro-organisms and extracellular matrices produced by these microorganisms, and their formation contributes to antimicrobial tolerance. In patients receiving central venous nutrition therapy via a central venous catheter, 15–37% of CRBSIs are polymicrobial infections [5,6]. Biofilms formed by multiple microbial species often exhibit different antimicrobial tolerance profiles compared with those of single-species biofilms. For instance, in dual-species biofilms of *Staphylococcus aureus* and *Candida albicans*, *S. aureus* shows increased vancomycin tolerance [7,8]. Similarly, in dual-species biofilms of *Pseudomonas aeruginosa* and *C. albicans*, the tolerance of *P. aeruginosa* to meropenem (MEPM) is increased [9]. Our previous study demonstrated that *Escherichia coli* exhibits increased tolerance to MEPM within *E. coli-C. albicans* dual-species biofilms [10]. In device-related infections, the primary treatment option is device removal. However, this may be infeasible for critically ill or long-term catheterized patients. In such cases, biofilm formation by multiple microbial species complicates treatment. Therefore, understanding the effects of combined antimicrobial agents against dual-species biofilms is crucial for selecting effective empirical treatment strategies.

The efficacy of antimicrobial agents can differ between *in vitro* and *in vivo* conditions. In the body, drugs often bind to proteins, primarily albumin, which reduces their pharmacological activity since only the free-form drug inhibits pathogen growth [11]. In fact, the antibacterial effects of certain drugs are altered significantly in the presence of albumin or serum [11,12]. Therefore, the therapeutic effects of drugs with high protein-binding rates need to be evaluated *in vivo* [13]. Silkworms are a useful invertebrate model for assessing the therapeutic effects of antimicrobial agents against systemic infections caused by pathogenic micro-organisms [14,16]. Compared with mammals, such as mice, silkworms are cost-effective and easy to breed in large numbers in limited space and raise fewer ethical concerns, making them ideal for large-scale studies [14,16]. Their relatively large body size allows for precise administration of test samples into haemolymph [14,15]. We have previously developed a biofilm evaluation model by inserting a catheter-like substrate into the silkworm body and used the model to confirm the increased drug tolerance of dual-species biofilms [17].

In this study, we screened effective combination therapies against *E. coli-C. albicans* dual-species biofilms *in vitro* and verified the observed effects using a silkworm biofilm infection model.

## Methods

### Strains and preculture conditions

*E. coli* strain RB-3 isolated from a patient’s blood culture and *C. albicans* strain SC5314 were used in this study [10]. For *in vitro* experiments, *E. coli* was precultured in tryptic soy broth at 37 °C for 24 h at 150 r.p.m. For *in vivo* experiments, *E. coli* was cultured on nutrient agar at 37 °C for 24 h. *C. albicans* was pre-cultured on Sabouraud dextrose agar at 27 °C for 24 h.

### Antimicrobial reagents

MEPM and amphotericin B (AMPH-B) were purchased from FUJIFILM Wako Pure Chemical Corporation (Osaka, Japan), fluconazole (FLCZ) from Tokyo Chemical Industry Co., Ltd. (Tokyo, Japan), and micafungin (MCFG) from Sigma-Aldrich, Inc. (St. Louis, MO, USA). MEPM, AMPH-B and FLCZ were dissolved in DMSO, while MCFG was dissolved in distilled water. All antimicrobial reagents were stored at −30 °C for *in vitro* experiments, and for *in vivo* experiments, reagents were dissolved in physiological saline (0.9% NaCl).

### Antimicrobial susceptibility testing of dual-species biofilms *in vitro*

Biofilms were grown in flat-bottomed 96-well microtitre plates as described previously [10]. Pre-cultures of *E. coli* were centrifuged, and the pellets were washed with PBS. *E. coli* and *C. albicans* were diluted in RPMI 1640 medium supplemented with MOPS (pH 7.3) to A_630_ values of 0.0001 and 0.1, respectively. The cell suspensions were incubated in flat-bottomed 96-well microtitre plates at 37 °C for 24 h. After biofilm formation, non-adherent cells were removed, and fresh medium containing antimicrobial reagents at the appropriate concentrations or equivalent solvent was added. After an additional 24-h incubation, the supernatant was discarded, and the cells were washed three times with PBS. Biofilms were then resuspended by vigorous pipetting, and appropriately diluted suspensions were plated on nutrient agar medium containing MCFG (for *E. coli* counting) and Sabouraud agar medium containing streptomycin (for *C. albicans* counting). After 24–48 h of incubation, the c.f.u. values of *E. coli* and *C. albicans* were counted.

### Silkworm rearing

Silkworms were reared as described previously [17]. Silkworm eggs were purchased from Ehime-Sanshu Co., Ltd. (Ehime, Japan), disinfected and hatched. The larvae were maintained at 25–27 °C and fed Silkmate 2S artificial diet (Ehime-Sanshu Co., Ltd.) mixed with vancomycin (300 µg g^−1^) until they reached the first-instar stage. Fourth-instar larvae were fed Silkmate 2S, while fifth-instar larvae, used for assays, were fed an antibiotic-free artificial diet (Sysmex Corporation, Hyogo, Japan) to avoid the impact on the growth of *E. coli* for 24 h.

### Antimicrobial susceptibility testing of dual-species biofilms in the silkworm model

Dual-species biofilm assays in the silkworm model were performed as described previously [17]. Polyurethane fibres (PFs; thickness: 0.5 mm, Gomutegusu F046, No. H3; Daiso Industries, Co., Ltd., Hiroshima, Japan) were cut into 1.5 cm pieces, sterilized with 70% ethanol and UV irradiation and inserted into the silkworms from their back. Once bleeding stopped, suspensions of *E. coli* and *C. albicans* in physiological saline were injected into the PF-inserted silkworms, followed by incubation at 27 °C for 18 h. Antimicrobial solutions were injected, and the PF was removed after 1 h. The biofilm cells on the PF were resuspended in physiological saline by vortexing for 15 min. Appropriate dilutions were plated on selective agar media, similar to the *in vitro* experiment, and the c.f.u. values of *E. coli* and *C. albicans* were counted.

### Statistical analysis

Viable cells in single and combination treatments in the silkworm model assay were compared using Student’s t-tests. A *P*-value of <0.05 was considered statistically significant.

## Results

### AMPH-B enhances the effect of MEPM against *E. coli*-***C.** albicans* dual-species biofilms

In *E. coli-C. albicans* dual-species biofilms, *E. coli* exhibited increased tolerance to MEPM, and the culture supernatant of *C. albicans* induced tolerance [10], suggesting that the presence of *C. albicans* in the biofilm environment influenced * E. coli* tolerance. We hypothesized that suppressing *C. albicans* within the biofilm would eliminate this effect and therefore investigated the effects of a combination of MEPM and antifungal agents.

**Fig. 1.**
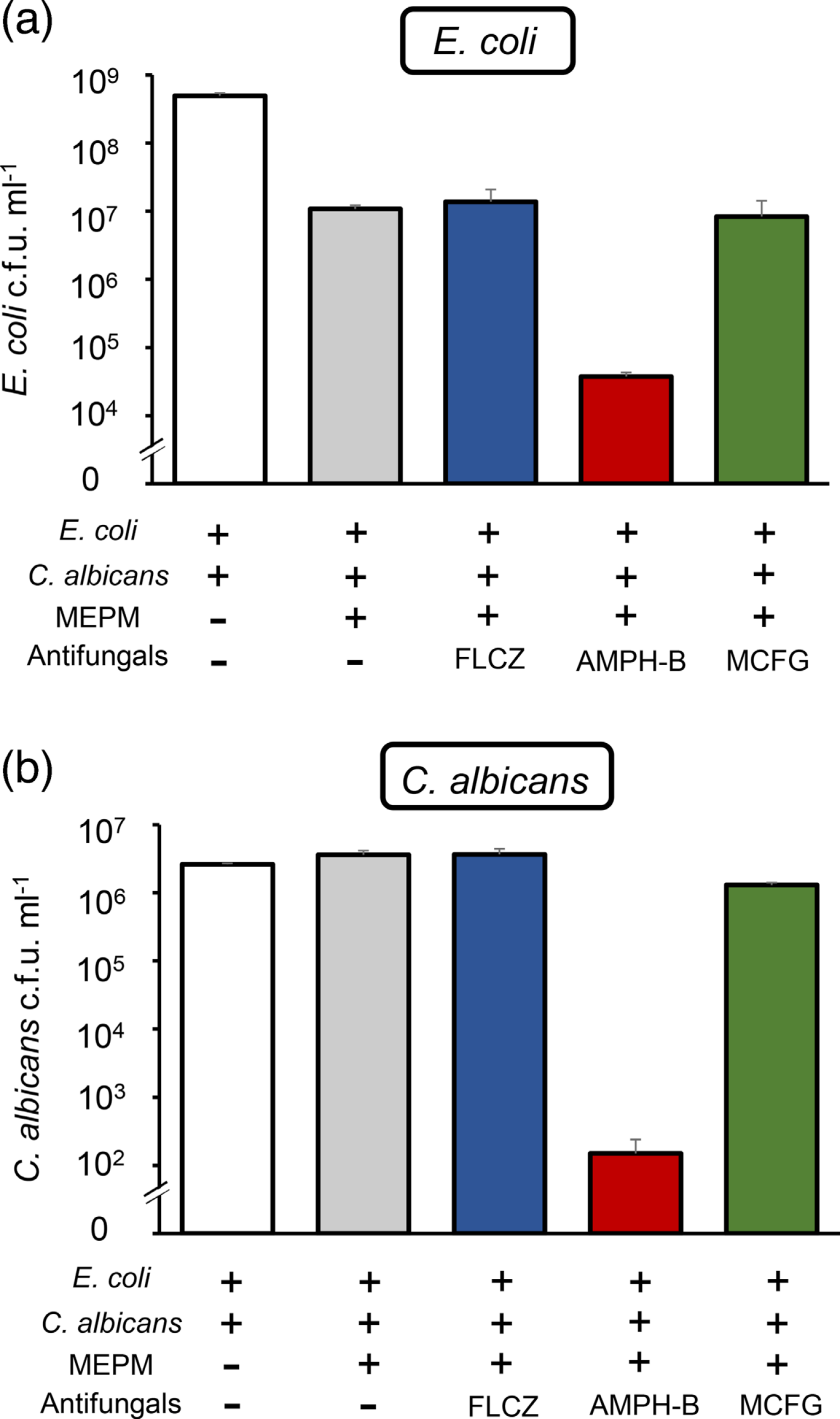
AMPH-B is the most effective agent in combination with MEPM against *E. coli*-*C. albicans* dual-species biofilms. Dual-species biofilms were treated with no antimicrobial drugs, only MEPM (50 µg ml^−1^) or a combination of MEPM (50 µg ml^−1^) and antifungals such as FLCZ, AMPH-B or MCFG (10 µg ml^−1^). The survival of *E. coli* (**a**) and *C. albicans* (**b**) was quantified by selective plating. Data are presented as means±sd from three replicates.

FLCZ, AMPH-B and MCFG, commonly used to treat candidemia, were tested at 10 µg ml^−1^ each. Compared with viability estimates under MEPM alone, the combination of MEPM with FLCZ did not reduce the viable cell counts of either *E. coli* or *C. albicans*. In contrast, the combination of MEPM with AMPH-B resulted in an ~280-fold reduction in *E. coli* and a 24,000-fold reduction in *C. albicans* compared with cell counts under MEPM alone. The combination of MEPM with MCFG reduced the *C. albicans* viable cell count by ~3-fold; however, the reduction in *E. coli* was minimal (1.3-fold; Fig. 1a, b).

**Fig. 2.**
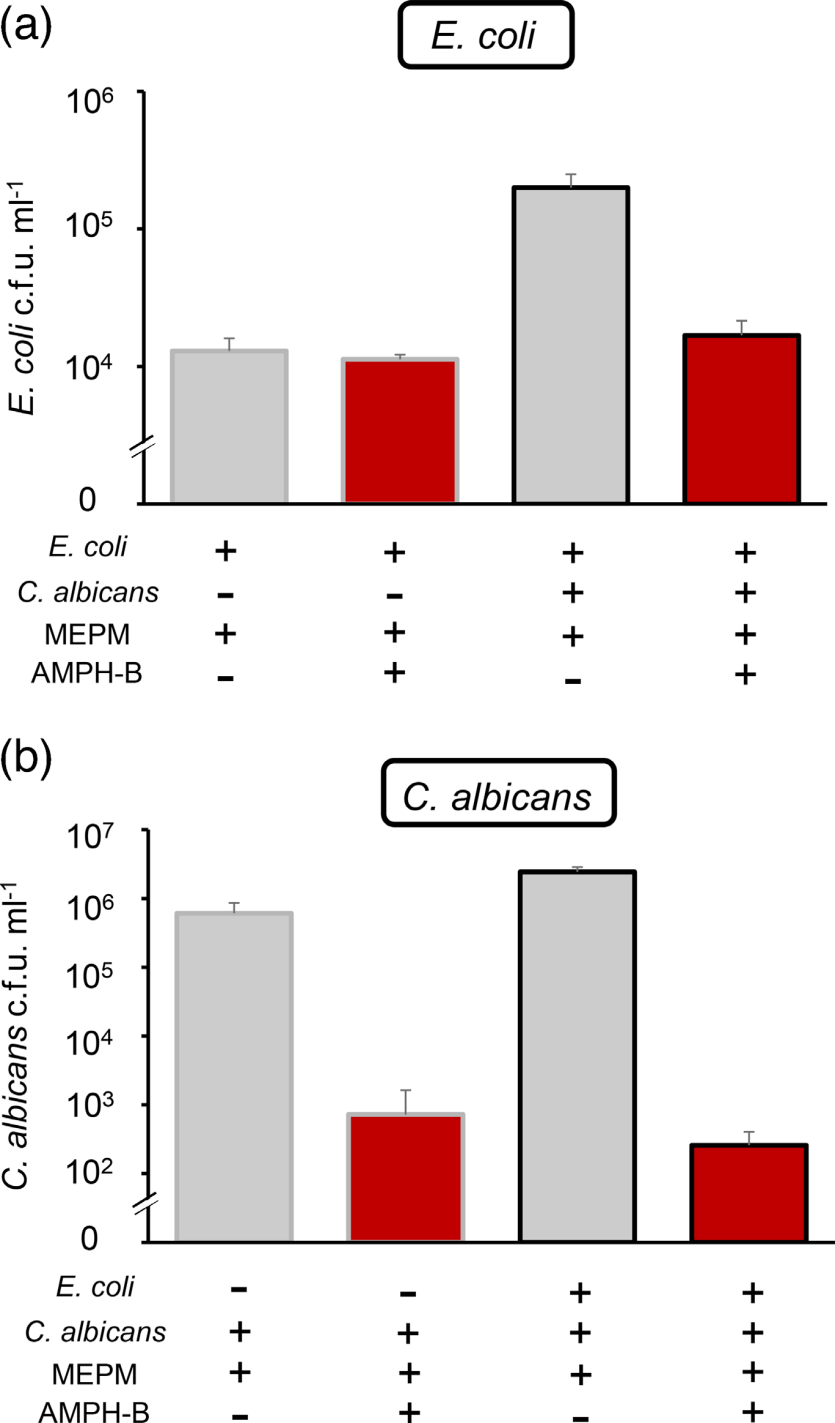
Combination effect of MEPM and AMPH-B against single- or dual-species biofilms. Single- and dual-species biofilms were treated with only MEPM (50 µg ml^−1^) or a combination of MEPM (50 µg ml^−1^) and AMPH-B (10 µg ml^−1^). The survival of *E. coli* (**a**) and *C. albicans* (**b**) was quantified by selective plating. Data are presented as means±sd from three replicates.

We focused on the most effective combination (MEPM+AMPH-B) in the further analyses of single-species and dual-species biofilms of *E. coli* and *C. albicans*. In single-species *E. coli* biofilms, no difference in the viable cell count was observed between MEPM alone and the combination of MEPM with AMPH-B. Compared with that of single-species *E. coli* biofilms, in dual-species biofilms, *E. coli* exhibited an increased tolerance. However, the combination of MEPM and AMPH-B reduced the *E. coli* viable cell count to levels comparable to those observed with MEPM in single-species *E. coli* biofilms (Fig. 2a).

**Fig. 3.**
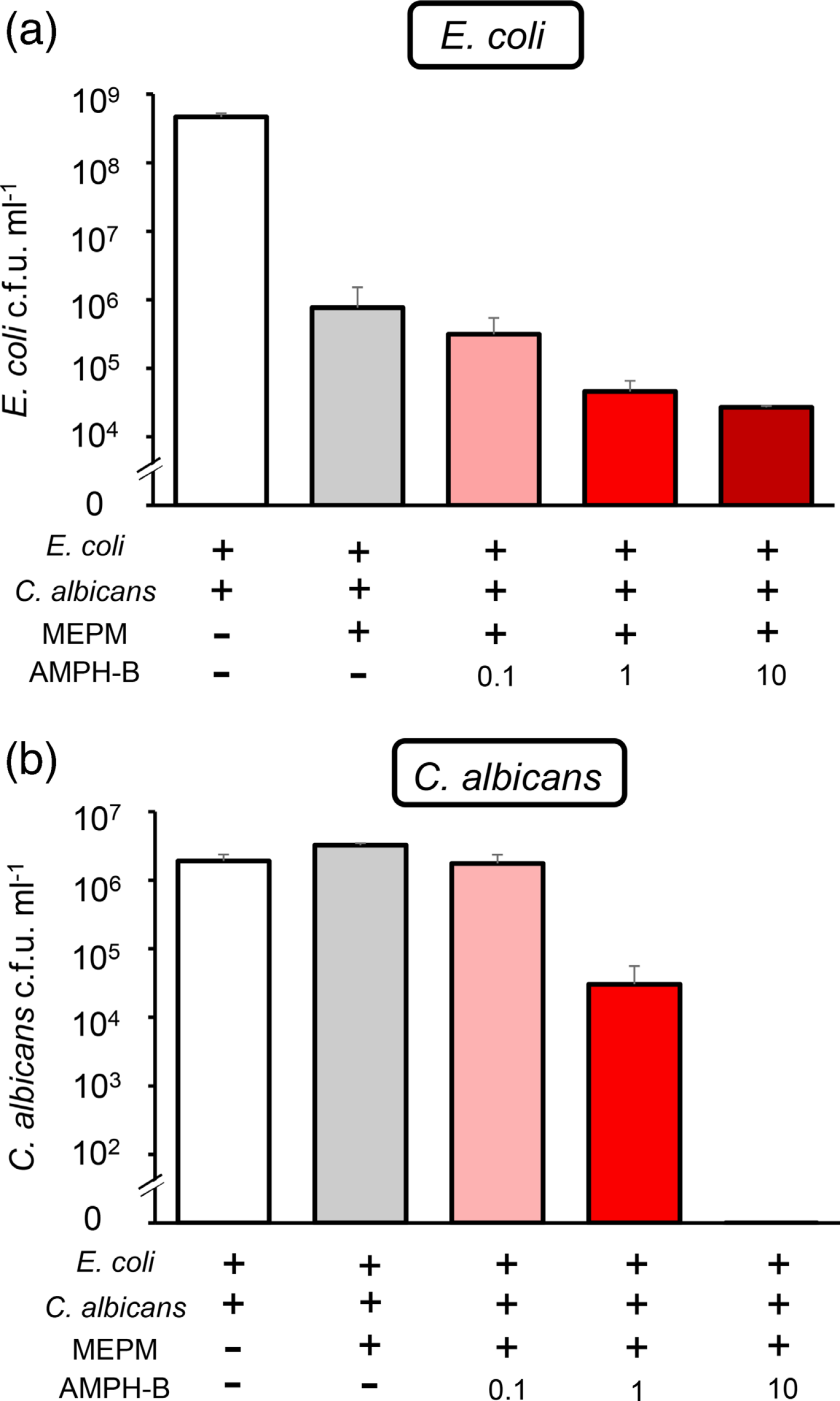
AMPH-B concentration-dependent increase in MEPM susceptibility of *E. coli* in dual-species biofilms. Dual-species biofilms were treated with no antimicrobial drugs, only MEPM (50 µg ml^−1^) or a combination of MEPM (50 µg ml^−1^) and AMPH-B (0.1, 1 or 10 µg ml^−1^). The survival of *E. coli* (**a**) and *C. albicans* (**b**) was quantified by selective plating. Data are presented as the means±sd from three replicates.

For *C. albicans*, AMPH-B reduced the viable cell counts in both single- and dual-species biofilms (Fig. 2b). In dual-species biofilms, the reduction in the *C. albicans* viable cell count was dependent on the AMPH-B concentration and was accompanied by a reduction in the *E. coli* viable cell count (Fig. 3a, b).

### Efficacy of MEPM and AMPH-B administration against *E. coli*-*C. albicans* dual-species biofilms in silkworms

In our *in vitro* studies, among the antifungal agents tested, AMPH-B exhibited the strongest inhibitory effect against dual-species biofilms when combined with MEPM. Therefore, we evaluated this combination therapy using a silkworm-based biofilm model.

Silkworm larvae implanted with a PF were inoculated with *E. coli* (6.5–8.1×10⁸ c.f.u./larva) and *C. albicans* (1.5–1.8×10⁶ c.f.u./larva) and incubated for 18 h. The larvae were then treated with MEPM alone (250 µg/larva) or in combination of MEPM with FLCZ or AMPH-B (25 µg/larva), and the viable cell counts in the biofilm were measured.

**Fig. 4.**
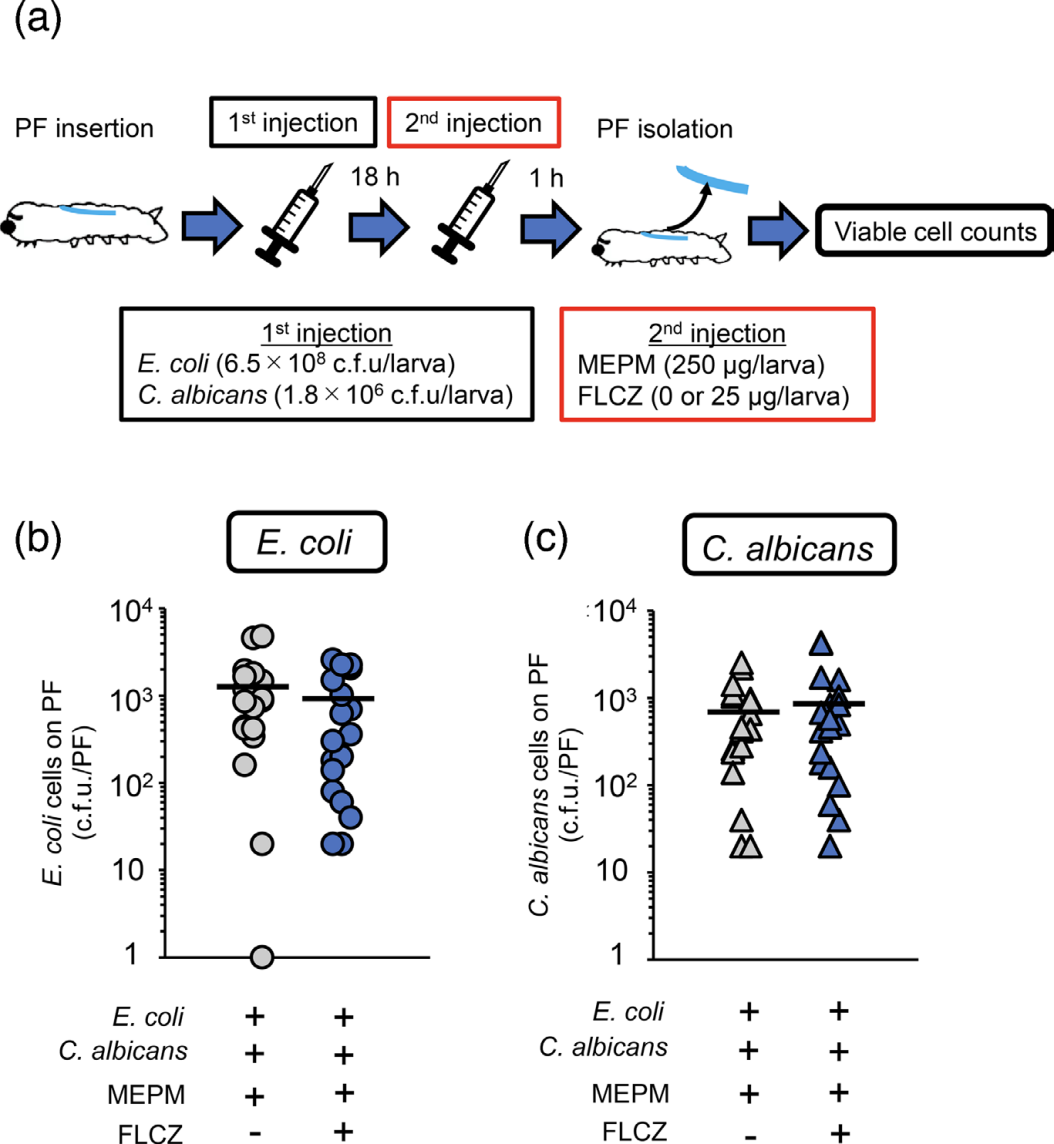
FLCZ does not affect MEPM tolerance of *E. coli* in a dual-species biofilm on the surface of PFs in silkworms. (**a**) Experimental scheme. Cell suspensions (*E. coli*: 6.5×10^8^ cells and *C. albicans*: 1.8×10^6^ cells/50 µl) were injected into PF-inserted silkworms, and the infected silkworms were incubated at 27 °C. After 18 h of incubation, only MEPM solution (250 µg/50 µl) or a mixed drug solution (MEPM: 250 µg and FLCZ: 25 µg/50 µl) was administered, and the silkworms were incubated at 27 °C for 1 h. (**b, c**) Viable *E. coli* cells (**b**) and *C. albicans* cells (**c**) on the PF surface in silkworms were measured. *n*=20/group. Significant differences between groups were evaluated using Student’s t-tests. *P<0.05.

The combination of MEPM and FLCZ, which did not show a suppressive effect against dual-species biofilms *in vitro*, resulted in viable cell counts of *E. coli* and *C. albicans* of ~10³ c.f.u., with no significant difference between MEPM alone and MEPM with FLCZ (Fig. 4).

**Fig. 5.**
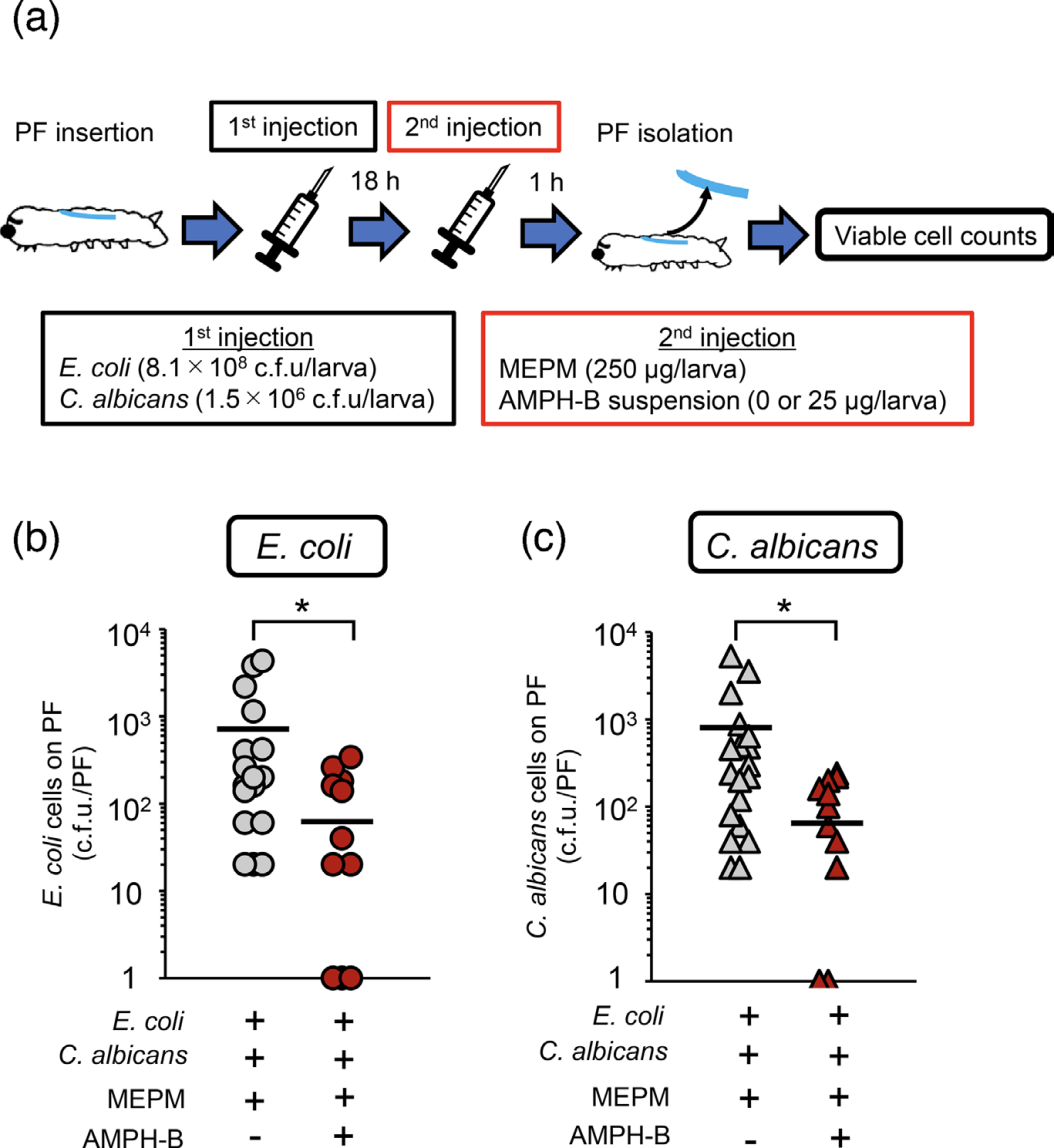
AMPH-B decreases MEPM tolerance of *E. coli* in a dual-species biofilm on the surface of PFs in silkworms. (**a**) Experimental scheme. Cell suspensions (*E. coli*: 8.1×10^8^ cells and *C. albicans*: 1.5×10^6^ cells/50 µl) were injected into PF-inserted silkworms, and the infected silkworms were incubated at 27 °C. After 18 h of incubation, only MEPM solution (250 µg/50 µl) or a mixed drug solution (MEPM: 250 µg and AMPH-B suspension: 25 µg/50 µl) was administered, and the silkworms were incubated at 27 °C for 1 h. (**b, c**) Viable *E. coli* cells (**b**) and *C. albicans* cells (**c**) on the PF surface in silkworms were measured. *n*=20/group. Significant differences between groups were evaluated using Student’s t-tests. *P<0.05.

In contrast, compared with MEPM alone, when MEPM was combined with AMPH-B, the viable cell counts of both *E. coli* and *C. albicans* decreased by 10-fold. A significant difference was observed between the MEPM alone and combination treatment groups (Fig. 5).

## Discussion

A previous study has demonstrated that *E. coli* exhibits increased tolerance to MEPM in *E. coli-C. albicans* dual-species *C. albicans* biofilms [10]. Therefore, the present study investigated effective combination therapies against these biofilms. FLCZ, which is a fungistatic drug that targets 14*α*-demethylase, is commonly used to treat candidiasis at doses ranging from 50 to 400 mg day^−1^, with sepsis guidelines recommending a target dose of 400 mg day^−1^ (with an initial dose of 800 mg day^−1^). A single administration of 400 mg results in a plasma concentration of ~17.5 µg ml^−1^ [18]. AMPH-B, which is a fungicidal drug that interacts with ergosterol molecules and forms a membrane-spanning pore, is approved for the treatment of fungal infections at doses ranging from 2.5 to 5 mg kg^−1^ (≥6 mg kg^−1^ for cryptococcosis), with a single dose of 2.5 mg kg^−1^ producing a maximum plasma concentration of 17.2 µg ml^−1^ [19]. MCFG, which is a fungicidal drug that inhibits *β*-1,3-glucan synthase, indicated for candidiasis, is administered at doses of 50–300 mg day^−1^, with a recommended dose of 100 mg day^−1^ for sepsis. A single 75 mg dose results in plasma concentrations of ~8 µg ml^−1^, whereas 150 mg results in 14 µg ml^−1^ [20]. Since the maximum plasma concentrations of these antifungal agents are ~10 µg ml^−1^, all experiments in this study were conducted at this concentration.

The combination of MEPM and FLCZ did not reduce the viable cell counts of *E. coli* or *C. albicans* in dual-species biofilms. Antifungal agents, particularly azoles, generally exhibit lower efficacies against biofilms than against planktonic cells, with a substantial difference for FLCZ [21]. This tolerance is caused by multiple factors including inhibition of penetration due to the extracellular matrix and increased cell density, expression of drug efflux pumps and the presence of persister cells. This aligns with our findings and those of previous reports, suggesting that FLCZ is ineffective against *C. albicans* in dual-species biofilms and, consequently, had no impact on *E. coli* viability.

In contrast, the combination of MEPM with MCFG resulted in a threefold reduction in the *C. albicans* viable cell count compared with that in the control (no drug). Previous studies have reported 50–75% inhibition of *C. albicans* mature biofilms by MCFG [22,23], consistent with our results. While MCFG inhibited *C. albicans* in dual-species biofilms, the viable cell counts of *E. coli* remained largely unchanged, suggesting that a threefold reduction in *C. albicans* was insufficient to significantly affect *E. coli* tolerance to MEPM.

When MEPM was combined with AMPH-B, the viable cell count of *C. albicans* decreased significantly. AMPH-B has been reported to inhibit over 95% of *C. albicans* mature biofilms [24,25], consistent with our findings. Additionally, the *E. coli* viable cell count decreased by >10-fold. Although some antifungal agents possess antibacterial activity [26], AMPH-B does not directly inhibit *E. coli*. Indeed, in single-species *E. coli* biofilms, no difference was observed between the effects of administering the combination of MEPM and AMPH-B and administering MEPM alone. Therefore, the reduction in the *E. coli* viable cell count in dual-species biofilms was due to a decrease in *C. albicans*. Additionally, the increased MEPM tolerance in *E. coli* was induced by the presence of *C. albicans*, and once *C. albicans* was significantly reduced, *E. coli* tolerance returned to the levels observed in single-species biofilms. Moreover, experiments with varying concentrations of AMPH-B showed that as the *C. albicans* viable cell count decreased in a concentration-dependent manner, the *E. coli* viable cell count also decreased. The adhesion of bacterial cells to the hyphae of *C. albicans* is critical for biofilm formation by some bacterial species. In *C. albicans-S. aureus* biofilms, eliminating *C. albicans* with AMPH-B alone reduces the *S. aureus* viable cell count, even without an antibacterial agent. However, this phenomenon was not observed in *E. coli-C. albicans* biofilms [27]. In addition, because a previous study showed that MEPM tolerance was induced in *E. coli* single biofilms by the supernatant of *C. albicans* [10], we believe that the effect is not due to the adhesion of *E. coli* to *C. albicans* hyphae, but rather due to components secreted or utilized by viable *C. albicans* cells. We speculated that the results of this study are not caused by changes in the hyphal cell structure, but rather by the death of *C. albicans* cells. Thus, our findings suggest that combination therapy with both antifungal and antibacterial agents is necessary for treating * E. coli-C. albicans* dual-species biofilms, with AMPH-B being the most effective antifungal option.

Subsequently, we investigated whether the *in vitro* results could be replicated *in vivo* using a silkworm-based biofilm evaluation system established in our previous study. In the model, the combination of MEPM and MCFG did not reduce the viable cell counts of *E. coli* or *C. albicans* [17]. Therefore, we evaluated the *in vivo* effects of the combination of MEPM with AMPH-B, which reduced viable cell counts *in vitro*, and the combination of MEPM with FLCZ, which did not. MEPM was administered at 250 µg/larva, a dose that previously demonstrated a reduction of viable cell counts in single-species biofilms and had no effect on dual-species biofilms. For AMPH-B, a dose of 25 µg/larva was selected. Considering that a silkworm weighs ~2 g and has ~500 µl of haemolymph, 25 µg/larva of AMPH-B was administered to achieve a concentration of 50 µg ml^−1^ in silkworms; this concentration is comparable to the levels observed in patients with febrile neutropenia (maximum plasma concentration of 57.6 µg ml^−1^ at 5 mg kg^−1^) [19]. Even though 50 µg ml^−1^ FLCZ in silkworm is considerably higher than the maximum human plasma concentration of ~8 µg ml^−1^, FLCZ was administered at a dose of 25 µg/larva for direct comparison with AMPH-B.

In the FLCZ combination group (MEPM+FLCZ), viable cell counts on the catheter fibres extracted from silkworms remained unchanged compared with those in the MEPM alone group for both *E. coli* and *C. albicans*. In contrast, in the AMPH-B combination group (MEPM+AMPH-B), the viable cell counts of both *E. coli* and *C. albicans* were significantly lower than those in the MEPM alone group. These *in vivo* results were consistent with the findings of the *in vitro* experiments, supporting the efficacy of the MEPM+AMPH-B combination. Considering that AMPH-B has a high protein-binding rate, which can lead to discrepancies between *in vitro* and physiological conditions [28], the observed effect of the combination in silkworms strongly suggests that this therapy will also be effective in clinical settings.

This study demonstrated that the combination of MEPM and AMPH-B was effective against *E. coli-C. albicans* dual-species biofilms both *in vitro* and *in vivo*. Since the *in vivo* model was based on silkworms, further validation in mammalian models is necessary. Nonetheless, our findings suggest that in cases where catheter removal is unfeasible in catheter-related * C. albicans-E. coli* co-infection, combination therapy with AMPH-B and antibacterial agents is a viable treatment strategy.

## Abbreviations

AMPH-B: amphotericin B
BSI: bloodstream infection
CRBSIs: catheter-related bloodstream infections
FLCZ: fluconazole
MCFG: micafungin
MEPM: meropenem
PF: polyurethane fibre
